# Analgesic effects of intraarticular anaesthetic lidocaine and methylprednisolone versus methylprednisolone alone following knee arthroscopy

**DOI:** 10.5599/admet.2412

**Published:** 2024-08-03

**Authors:** Wahid Mohammed Hassan, Anas Amer Mohammad

**Affiliations:** University of Duhok, Duhok, Iraq

**Keywords:** Analgesic, postoperatively, intraarticular, intraarticular, methylprednisolone

## Abstract

**Background:**

Knee arthroscopy is a widely practiced orthopaedic procedure known for its minimally invasive approach, allowing quicker recovery times and less postoperative discomfort than traditional open surgeries. However, managing postoperative pain remains a critical aspect of patient care and satisfaction. The main objective of this research is to examine the relationships between patient demographics (age, gender, BMI) and early postoperative outcomes, including pain, physiotherapy, and walking.

**Method:**

Randomized data collection, clinical trial study of 2 groups of patients. The patients were split into lidocaine 1 % 16 ml + methylprednisolone 160 mg 4 ml) and (methylprednisolone only 160 mg 4 ml) groups. All patients in both groups were queried about age, gender, BMI, and pain on the first, third, and 15th days following surgery. All patients were tested for physiotherapy on the second, third, and fourth postop days. After surgery, walking was tested on the third, fourth, and fifth days.

**Results:**

Significant differences in postoperative pain relief and physiotherapy initiation times were observed. There are notable associations between treatment groups and recovery metrics, such as pain levels and mobility on various days’ post-surgery. Significant demographic influences (age, gender, BMI) on recovery outcomes are observed, particularly in walking and pain at day 15 post-operation.

**Conclusion:**

lidocaine and methylprednisolone improve postoperative pain relief and functional recovery in knee arthroscopy patients, with most experiencing reduced pain early post-surgery (early physiotherapy) and an expedited return to walking (decreased morbidity). Patients taking just methylprednisolone recovered slower. Age, gender, and BMI affected pain and walking abilities post-operation but not physiotherapy time, underscoring the personalised approach needed in postoperative treatment.

## Introduction

Knee arthroscopy is a widely practiced orthopaedic procedure known for its minimally invasive approach, allowing quicker recovery times and less postoperative discomfort than traditional open surgeries. However, managing postoperative pain remains critical to patient care and satisfaction [1,2]. The introduction of intra-articular anaesthetic injections, specifically the combination of methylprednisolone and lidocaine, represents a significant advancement in postoperative pain management strategies [3,4]. This method combines the anti-inflammatory benefits of a corticosteroid with the immediate analgesic effects of a local aesthetic, providing a dual-action approach to control pain directly at the surgical site. This aligns with the principles of multimodal analgesia, aiming to reduce reliance on opioids and minimize their side effects [5].

The efficacy of methylprednisolone and lidocaine injections for postoperative pain relief is supported by a growing body of research, with studies published in reputable medical journals highlighting their role in reducing pain and facilitating early functional recovery. These findings are crucial for improving patient outcomes, offering targeted pain relief, and mitigating the inflammatory response induced by surgery [6]. However, the implementation of this treatment must be approached with caution due to potential risks such as cartilage toxicity and infection. Thus, patient selection and dosage optimization are key considerations, underlining the importance of evidence-based treatment protocols and ongoing research to refine their use [7]. Knee arthroscopy's popularity [8] as a procedure stem from its ability to be performed on an outpatient basis, with patients rarely requiring hospitalization before or after surgery. Despite the advantages of arthroscopy, postoperative pain can vary significantly among patients, necessitating various analgesia strategies. These include systemic drugs like NSAIDs, central and peripheral blocks, and the intra-articular administration of drugs, which has been shown to significantly reduce pain and decrease the need for additional analgesics [9,10]. Intra-articular injections have been explored with various agents, including local anaesthetics like lidocaine and bupivacaine, opiates such as morphine and fentanyl, benzodiazepines like midazolam, α2-agonists such as clonidine and dexmedetomidine, and even magnesium sulphate. These agents, alone or in combination, have been demonstrated to provide effective analgesia, sometimes lasting up to 24 hours and potentially reducing chronic pain [11,12]. The mechanisms of action for these drugs include blocking the transmission of action potentials through the inhibition of sodium channels by local aesthetics and modulating peripheral opiate receptors, which proliferate during inflammation in peripheral tissues [13,14]. Additionally, the analgesic effects of midazolam are attributed to its agonistic action on benzodiazepine peripheral receptors and GABA receptors, indicating a broad potential for various agents in managing postoperative pain through intra-articular administration [15].

The aim of this study is to investigate the effects of intra-articular anaesthetic injection, specifically comparing the combination of lidocaine and methylprednisolone versus methylprednisolone alone, on postoperative pain management, functional recovery, and the time for physiotherapy following knee arthroscopy. Additionally, it seeks to explore the associations between patient demographics (age, gender, BMI) and postoperative outcomes, including pain levels, the initiation of physiotherapy, and the ability to commence walking during the early postoperative period.

## Method

Randomized data collection, clinical trial study of 2 groups of patients (65) underwent knee arthroscopy in Duhok emergency hospital and the data collected from March 2023 to March 2024. The patients were divided into 2 groups: (lidocaine 1 % 16 ml + methylprednisolone 160 mg 4 ml) and (methylprednisolone only 160 mg 4 ml). All patients in both groups were asked about age, gender, and body mass index (BMI). All patients were assessed for pain on the 1st, 3rd and 15th day after operation. Also, all patients were assessed for starting physiotherapy on 2^nd^, 3^rd^ and 4^th^-day post-operation. Walking was also assessed on 3^rd^, 4^th^ and 5^th^-day post-operation. The statistical analysis was carried out using SPSS version 22, employing frequency and percentage for categorical data and mean and standard deviation (SD) for continuous data. The chi-square test was utilized to determine the association between categorical variables, *P*-value of 0.05 or lower was considered statistically significant. The research was approved by the Ethics committee of the University of Duhok.

## Results and discussion

Table 1 shows the age of patients who received lidocaine + methylprednisolone. 52.2 % were ≥30 years old, 54.5 % of patients who received lidocaine + methylprednisolone were females. 85.7 % of patients who received lidocaine + methylprednisolone was obese. The mean age of patients who received lidocaine + methylprednisolone was 29.2 years. While the mean age of patients who received methylprednisolone was 28 years, only 47.8 % of them were ≥30 years old, 45.5 % of patients who received (methylprednisolone only) were females. 14.3 % of patients who received (methylprednisolone only) were obese.

**Table 1.** Distribution of patients in both groups according to study variables.

Patients received		Number of patients		
		Lidocaine + methylrednisolone	Methylprednisolone only	Total
Age	<30	21 (50.0 %)	21 (50.0 %)	42
	30 and more	12 (52.2 %)	11 (47.8 %)	23
Gender	Females	12 (54.5 %)	10 (45.5 %)	22
	Males	21 (48.8 %)	225 (1.2 %)	43
Body mass index	Low	1 (50.0 %)	1 (50.0 %)	2
	Normal	9 (36.0 %)	16 (64.0 %)	25
	Overweight	17 (54.8 %)	14 (45.2 %)	31
	Obese	6 (85.7 %)	1 (14.3 %)	7

An interesting discovery was made: a connection worth noting is the (lidocaine + methylprednisolone) group and mild pain (93.9 %) of patients on the first day after operation. Another remarkable association is between the same group and being completely free from pain (84.8 %) on the 15^th^ day post-surgery. Equally important, there is no association between both groups and pain on 3^rd^ day post-operation, as shown in Table 2.

**Table 2.** Association between pain analysis according to study groups.

Variables	Pain analysis	Groups		*P*-value
		Lidocaine + methylprednisolone	Methylprednisolone only	
1^st^ day pain	Mild	31 (93.9 %)	0 (0.0%)	0.0001
	Moderate	2 (6.1 %)	9 (28.1 %)	
	Severe	0 (0.0 %)	23 (71.9 %)	
3^rd^ day pain	No	10 (30.3 %)	14 (43.8 %)	0.08
	Mild	18 (54.5 %)	17 (53.1 %)	
	Moderate	5 (15.2 %)	0 (0.0 %)	
15^th^ day pain	Severe	0 (0.0 %)	1 (3.1 %)	0.04
	No	28 (84.8 %)	20 (62.5 %)	
	Mild	4 (12.1 %)	12 (37.5 %)	
	Moderate	1 (3.0 %)	0 (0.0 %)	
	Total	33 (100.0 %)	32 (100.0 %)	

*P*-value ≤0.05 (significant)

There is a significant association between the (lidocaine + methylprednisolone) group and the time for physiotherapy (51.5 %) of patients started on the 2^nd^ day after the operation, while no patients started physiotherapy on the 4^th^-day postop. In the group (methylprednisolone only), 81.3 % of patients started physiotherapy on 3^rd^ day post-operation.

There is a significant association between the (lidocaine + methylprednisolone) group starting walking (81.8 %) of patients started to walk on 3^rd^ day after the operation, while in the group (methylprednisolone only) 40.6 % of patients started to walk on the 4^th^ day after the operation, as shown in Table 3.

**Table 3.** Distribution of patients in both groups according to studied variables.

Variables	Pain analysis	Groups		*P*-value
		Lidocaine + methylrednisolone	Methylprednisolone only	
Starting of physiotherapy	2^nd^ day	17 (51.5 %)	4 (12.5 %)	0.002
	3^rd^ day	16 (48.5 %)	26 (81.3 %)	
	4^th^ day	0 (0.0 %)	2 (6.3 %)	
Starting of walking	3^rd^ day	27 (81.8 %)	10 (31.3 %)	0.0001
	4^th^ day	4 (12.1 %)	13 (40.6 %)	
	5^th^ day	2 (6.1 %)	9 (28.1 %)	
	Total	33 (100.0 %)	32 (100.0 %)	

*P*-value ≤0.05 (significant)

### Group of patients received lidocaine + methylprednisolone

There is a significant association between age group and pain at day 15 post-operation, with 100 % of patients with mild and moderate pain in the age group ≥30 years. There is a significant association between gender, BMI and pain at day 15 post-operation, as shown in Table 4.

**Table 4.** Association between pain at day 15 post-operation and age groups, gender, BMI.

Variables		Pain at day 15 post operation			*P*-value
		No	Mild	Moderate	
Age group (years)	<30	21 (75.0 %)	0 (0.0 %)	0 (0.0 %)	0.006
	30 and more	7 (25.0 %)	4 (100.0 %)	1 (100.0 %)	
Gender	Females	8 (28.6%)	3 (75.0 %)	1 (100.0 %)	0.08
	Males	20 (71.4%)	1 (25.0 %)	0 (0.0 %)	
Body mass index	Low	1 (3.6%)	0 (0.0 %)	0 (0.0 %)	0.4
	Normal	7 (25.0%)	2 (50.0 %)	0 (0.0 %)	
	Overweight	16 (57.1%)	0 (0.0 %)	1 (100.0 %)	
	Obese	4 (14.3%)	2 (50.0 %)	0 (0.0 %)	
	Total	28 (100.0%)	4 (100.0 %)	1 (100.0 %)	

*P*-value ≤ 0.05 (significant)

Age group, gender and BMI are highly connected with initiating walking. All patients who began walking on the 4th day post-operation were aged ≥30 years. The females made up 100 % of those who initiated walking on the 5^th^ day post-operation, whereas the males constituted 74.1 % of those who started walking on the 4^th^ day post-operation. Being overweight was observed in 59.3 % of patients who started walking on the 3^rd^ day post-operation, while obesity was seen in 50 % of patients who started walking on the 4^th^ day post-operation, as shown in Table 5.

**Table 5.** Association between starting of walk and age groups, gender, BMI.

Variables		Starting of Walk			*P*-value
		3^rd^ day	4^th^ day	5^th^ day	
Age groups	<30	20 (74.1 %)	0 (0.0 %)	1 (50.0 %)	0.015
	30 and more	7 (25.9 %)	4 (100.0 %)	1 (50.0 %)	
Gender	Females	7 (25.9 %)	3 (75.0 %)	2 (100.0 %)	0.025
	Males	20 (74.1 %)	1 (25.0 %)	0 (0.0 %)	
Body mass index	Low	0 (0.0 %)	1 (25.0 %)	0 (0.0 %)	0.01
	Normal	7 (25.9 %)	0 (0.0 %)	2 (100.0 %)	
	Overweight	16 (59.3 %)	1 (25.0 %)	0 (0.0 %)	
	Obese	4 (14.8 %)	2 (50.0 %)	0 (0.0 %)	
	Total	27 (100.0 %)	4 (100.0 %)	2 (100.0 %)	

*P*-value ≤ 0.05 (significant)

There is no significant association between age group, gender and BMI, and starting of physiotherapy, as shown in Table 6.

**Table 6.** Association between age group, gender and BMI and starting of physiotherapy.

Variables		Starting of physiotherapy		
		2^nd^ day	3^rd^ day	*P*-value
Age groups	<30	11 (64.7 %)	10 (62.5 %)	1.000
	30 and more	6 (35.3 %)	6 (37.5 %)	
Gender	Females	6 (35.3 %)	6 (37.5 %)	1.000
	Males	11 (64.7 %)	10 (62.5 %)	
Body mass index	Low	1 (5.9 %)	0 (0.0 %)	0.5
	Normal	6 (35.3 %)	3 (18.8 %)	
	Overweight	7 (41.2 %)	10 (62.5 %)	
	Obese	3 (17.6 %)	3 (18.8 %)	
Total		17 (100.0 %)	16 (100.0 %)	

## Discussion

The discussion of these findings explores the significant associations observed between intra-articular injections following knee arthroscopy, patient demographics, and postoperative outcomes, including pain levels, physiotherapy requirements, and the ability to walk.

### Association between intra-articular injections and pain management

The significant reduction in pain observed on the first day post-operation in patients receiving a combination of lidocaine and methylprednisolone (93.9 %) compared to those receiving methylprednisolone alone highlights the enhanced analgesic effect of the combined treatment. This is consistent with the literature suggesting that the addition of a local aesthetic to corticosteroids can enhance pain relief by providing immediate and sustained analgesic effects [16]. The lack of significant difference in pain between groups on the third day post-operation suggests a transient benefit, with the most pronounced effects seen immediately after surgery. However, the substantial decrease in pain by the 15th day post-operation in the combination group (84.8 %) supports the long-term benefits of this approach. These findings align with [17], who reported similar outcomes in prolonged pain relief with combined intra-articular injections.

### Time for starting physiotherapy

The study's findings indicate a significant association between combination therapy and the time needed to start physiotherapy from the second day post-operation. This contrasts with the methylprednisolone-only group, where a higher percentage of patients required physiotherapy by the third day. This could suggest that effective pain management may facilitate earlier engagement in physical therapy, as patients experiencing less pain might be more willing to participate in rehabilitation exercises. This observation is supported by research indicating that early mobilization post-arthroscopy can improve functional outcomes [18]. However, the absence of a significant difference in the initiation of physiotherapy based on age, gender, and BMI suggests that the effectiveness of pain management strategies, rather than demographic factors, primarily influences the readiness for physiotherapy.

### Impact on the ability to walk

The significant improvement in the ability to walk by the third day post-operation in patients receiving the combined treatment (81.8 %) versus those receiving methylprednisolone alone (40.6 %) underscores the importance of effective pain management in early postoperative mobility. This finding is corroborated by studies indicating that reduced pain thresholds can significantly enhance patients' mobility and willingness to initiate walking post-surgery [19]. The association between demographic factors (age, gender, BMI) and the ability to walk suggests that these factors may influence recovery trajectories. Particularly, the findings that individual’s ≥30 years and males showed a quicker return to walking post-operation align with literature emphasizing the variability in recovery rates based on demographic and physiological differences [20].

### Limitations and further research

While the study provides valuable insights into the benefits of combined intra-articular injections for pain management and recovery post-knee arthroscopy, it also highlights the need for further research. The influence of demographic factors on recovery outcomes suggests that personalized approaches to postoperative care could optimize patient recovery. Further, longitudinal studies are necessary to understand the long-term impacts of these treatments on joint health, functionality, and quality of life.

## Conclusions

The study demonstrates that the combination of lidocaine and methylprednisolone significantly enhances postoperative pain relief and functional recovery in knee arthroscopy patients, with a majority experiencing reduced pain early post-surgery (early starting of physiotherapy) and an expedited return to walking (decrease morbidity). Contrastingly, patients receiving only methylprednisolone showed a delayed recovery. Demographic variables such as age, gender, and BMI were found to influence pain levels and the ability to start walking post-operation, yet did not significantly affect the time for physiotherapy, highlighting the tailored approach required in postoperative care.
